# Marked First Degree Atrioventricular Block: an extremely prolonged PR interval associated with Atrioventricular Dissociation in a young Nigerian man with Pseudo-Pacemaker Syndrome: a case report

**DOI:** 10.1186/1756-0500-7-781

**Published:** 2014-11-04

**Authors:** Oluwadare Ogunlade, Anthony O Akintomide, Olufemi E Ajayi, Omotayo A Eluwole

**Affiliations:** Department of Physiological Sciences, Obafemi Awolowo University, Ile-Ife, Nigeria; Department of Medicine, Obafemi Awolowo University, Ile-Ife, Nigeria; Department of Medical Pharmacology and Therapeutics, Obafemi Awolowo University, Ile-Ife, Nigeria

**Keywords:** Marked First Degree Atrioventricular Block, Extremely prolonged PR interval, Atrioventricular Dissociation, Pseudo-Pacemaker syndrome

## Abstract

**Background:**

The diagnosis of Marked First Degree Atrioventricular Block is made with electrocardiogram when PR interval ≥0.30 s. A PR interval of up to 0.48 s had been reported in literature. Data is sparse on an extremely prolonged PR interval associated with Atrioventricular Dissociation and Pseudo-Pacemaker Syndrome. Electrocardiogram with this type of uncommon features poses diagnostic and management challenges in clinical practice.

**Case presentation:**

We report a case of a 22 year old Nigerian male from Igbo ethnic group who presented himself for medical screening with a history of exercise intolerance, occasional palpitation and fainting spells. He has no history of cough, orthopnoea, paroxysmal nocturnal dyspnoea nor body swelling. A physical examination revealed that the patient has a pulse rate of 64 beats per minute, blood pressure of 110/70 mmHg and soft heart sounds. Standard 12-lead electrocardiogram showed an uncommon Marked First Degree Atrioventricular Block with an extremely prolonged PR interval of 0.56 s. Long rhythm strips of the electrocardiogram showed extremely prolonged PR interval associated with Atrioventricular Dissociation and variable degrees of Atrioventricular Block (Mobitz type I and II).

**Conclusions:**

An extremely prolonged PR interval may occur in First Degree Atrioventricular Block and it may be associated with Atrioventricular Dissociation and Pseudo-Pacemaker Syndrome which may pose diagnostic and management challenges. This suggests that not all cases of First Degree Atrioventricular Block are benign and so should be sub-classified based on degree of PR interval prolongation and associated electrical abnormalities.

## Background

Cardiovascular disorders are the leading causes of preventable deaths globally [1]. First Degree Atrioventricular (AV) Block characterized by prolongation of PR interval was initially taught to be a benign electrocardiographic finding [2]. The Framingham Heart Study (2009) reported that the presence of First Degree AV Block doubled the risk of developing atrial fibrillation, tripled the risk of requiring an artificial pacemaker and associated with a small increase in mortality. This risk was proportional to the degree of PR prolongation [3]. First Degree AV Block is diagnosed with electrocardiogram (ECG) when PR interval [measured from the onset of atrial depolarization (P wave) to the onset of ventricular depolarization (QRS complex)] is >0.20 s. Marked First Degree AV Block is defined when the PR interval ≥0.30 s [4]. It has been established that Marked First Degree AV Block can cause symptoms similar to those produced by Pacemaker Syndrome even without pacemaker and this can occur despite normal left ventricular function. This is what is referred to as Pseudo-Pacemaker Syndrome [5]. Extremely prolonged PR interval may produce unusual ECG features such as AV Dissociation, a common feature of Third Degree AV Block.

## Case presentation

**Figure 1:**
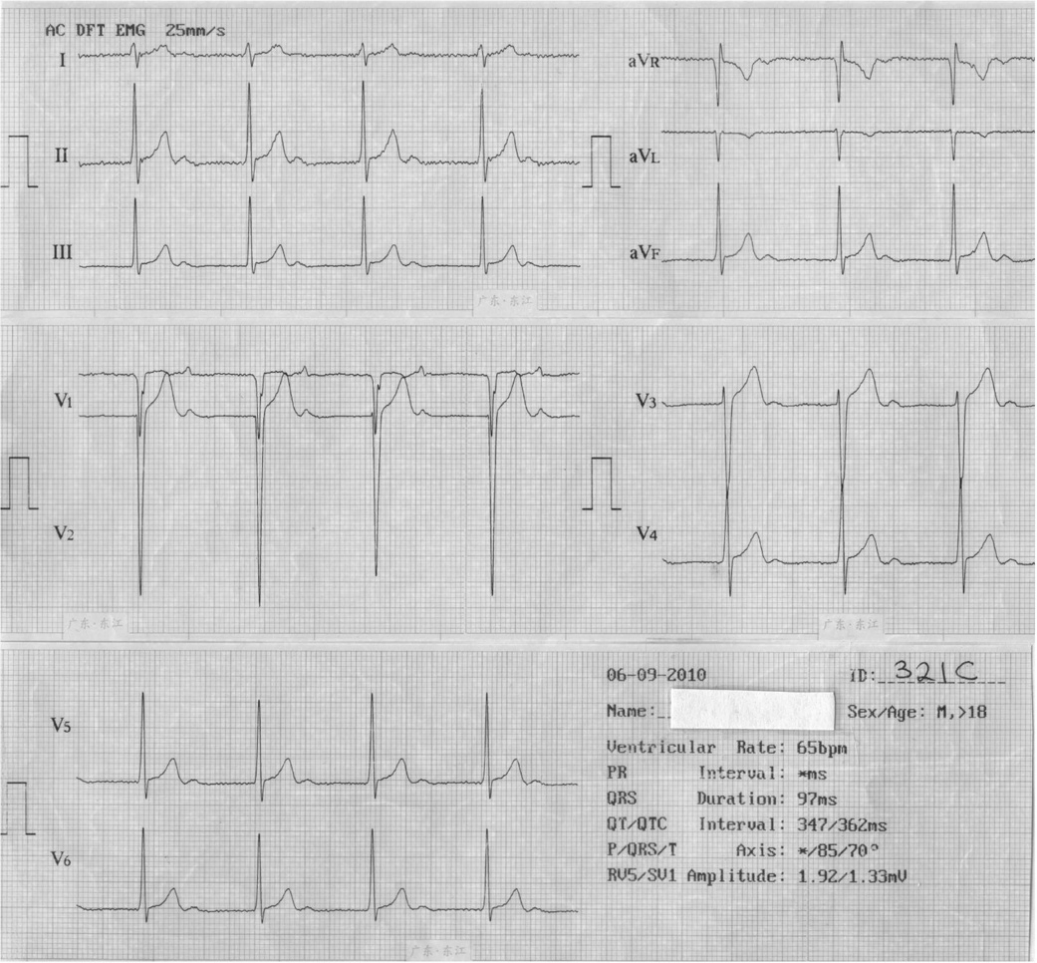
**A resting standard 12-lead electrocardiogram of a 22 year old man with a normal heart rate (65 beats per minute) and P wave occurring immediately after T wave with anextremely prolonged PR interval (0.56 s).** This was misinterpreted by the machine self-interpretation software as ‘absence’ of PR interval.

**Figure 2:**
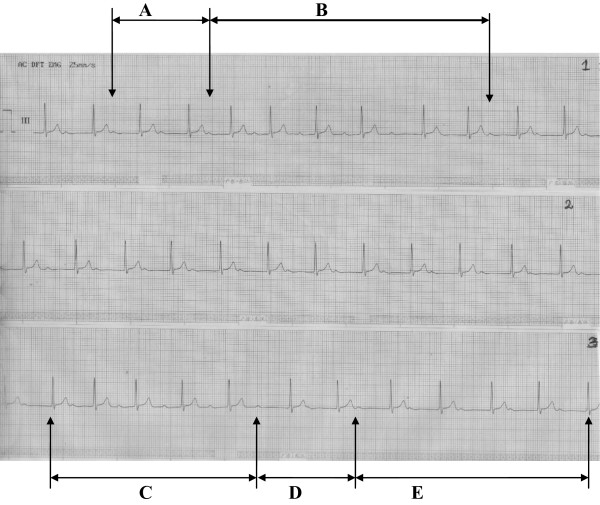
**Marked First Degree Atrioventricular Block with extremely prolonged PR interval (A) transiting to Atrioventricular Dissociation (B) in the first long rhythm strip (1).** The second long rhythm strip (2) maintained First Degree Atrioventricular Block throughout while the third long rhythm strip(3) commenced with First Degree Atrioventricular Block and progressed in it but with shortening of the PR interval **(C)** followed by Atrioventricular Dissociation **(D)** and a return to First Degree Atrioventricular Block with extremely prolonged PR interval **(E)**.

**Figure 3:**
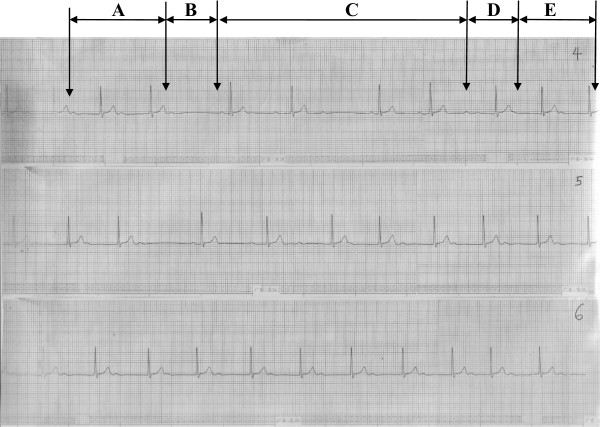
**Marked First Degree Atrioventricular Block with extremely prolonged PR interval (A) transiting into Mobitz type II(B), Mobitz type I(C), Atrioventricular Dissociation (D) and return to First Degree Atrioventricular Block (E) sequentially in fourth long rhythm strip (4).** Similar events occurred in the fifth long rhythm strip (**5)** but the sixth long rhythm strip **(6)** maintained First Degree Atrioventriccular Block throughout the length of the strip. Varying RR intervals occurred because of the presence of variable degrees of Atrioventricular Block.

A 22 year old Nigerian male from Igbo ethnic group presented himself for medical screening following recurrent history of exercise intolerance, occasional palpitation and fainting spells since early childhood. He has no history of cough, orthopnoea, paroxysmal nocturnal dyspnoea nor body swelling. A physical examination revealed that the patient has a pulse rate of 64 beats per minute, blood pressure of 110/70 mmHg and soft heart sounds. Standard 12-lead ECG showed an uncommon variable degree of AV block predominantly Marked First Degree AV block with an extremely prolonged PR interval of 0.56 s and AV Dissociation (Figures 1, 2 and 3). A clinical diagnosis of Pseudo-Pacemaker Syndrome was made and the patient was referred to the University affiliated Teaching Hospital for further investigations and management.

## Discussion

Marked First Degree AV Block with extremely prolonged PR interval ≥0.50 s is rare. Most reports on First Degree AV Block quote PR interval which are usually less than 0.50 s [5]. It has been established that PR interval ≥0.30 s should be classified as class IIa indication for pacing in a symptomatic individual [6]. With extremely prolonged PR interval, atrial systole occurs very close to preceding ventricular systole coupled with AV Dissociation. The consequential AV dyssynchrony results in haemodynamic disturbances which may be symptomatic and require a dual-chamber pacemaker insertion [6, 7]. The probability of fatal haemodynamic consequences may be high with an extremely prolonged PR interval with AV Dissociation like the index case. Therefore, sub-classification of Marked First Degree AV Block based on the extent of PR interval prolongation becomes imperative. Moreover, there is need for proper interpretation of every ECG by a cardiologist irrespective of the diagnosis made by the ECG self-interpretive software of the ECG machine. In this case, the machine self-interpreting software recorded ‘*’in place of PR interval. This was a misdiagnosis and could be misleading for an unsuspecting clinician and might have occurred because the machine self-interpreting software did not recognise a PR interval in the range of 0.56 s. First Degree AV Block should not just be termed a benign ECG finding but rather it should be properly interpreted and assessed based on clinical findings, degree of prolongation of the PR interval and associated electrical abnormalities such as AV Dissociation and variability of the AV Block.

## Conclusions

An extremely prolonged PR interval may occur in First Degree AV Block and it may be associated with Atrioventricular Dissociation and Pseudo-Pacemaker Syndrome which may pose diagnostic and management challenges. This suggests that not all cases of First Degree AV Blocks are benign and so should be sub-classified based on the degree of PR interval prolongation and associated electrical abnormalities.

## Consent

Written informed consent was obtained from the patient for publication of this case report and any accompanying images. A copy of the written consent is available for review by the Editor-in-Chief of this journal.

## Abbreviations

AV: Atrioventricular
ECG: Electrocardiogram
P wave: Atrial depolarization
QRS complex: Ventricular depolarization.
